# A putative UDP-glycosyltransferase from *Heterorhabditis bacteriophora* suppresses antimicrobial peptide gene expression and factors related to ecdysone signaling

**DOI:** 10.1038/s41598-020-69306-2

**Published:** 2020-07-23

**Authors:** Eric Kenney, Amulya Yaparla, John M. Hawdon, Damien M. O’Halloran, Leon Grayfer, Ioannis Eleftherianos

**Affiliations:** 1 Department of Biological Sciences, The George Washington University, Washington, DC 20052 USA; 2 Department of Microbiology, Immunology, and Tropical Medicine, The George Washington University, Washington, DC USA

**Keywords:** Biological techniques, Cancer, Microbiology, Immunology, Inflammation

## Abstract

Insect pathogens have adopted an array of mechanisms to subvert the immune pathways of their respective hosts. Suppression may occur directly at the level of host–pathogen interactions, for instance phagocytic capacity or phenoloxidase activation, or at the upstream signaling pathways that regulate these immune effectors. Insect pathogens of the family *Baculoviridae*, for example, are known to produce a UDP-glycosyltransferase (UGT) that negatively regulates ecdysone signaling. Normally, ecdysone positively regulates both molting and antimicrobial peptide production, so the inactivation of ecdysone by glycosylation results in a failure of host larvae to molt, and probably a reduced antimicrobial response. Here, we examine a putative ecdysteroid glycosyltransferase, Hba_07292 (*Hb-ugt-1*), which was previously identified in the hemolymph-activated transcriptome of the entomopathogenic nematode *Heterorhabditis bacteriophora*. Injection of recombinant *Hb-ugt-1* (r*Hb-ugt-1*) into *Drosophila melanogaster* flies resulted in diminished upregulation of antimicrobial peptides associated with both the Toll and Immune deficiency pathways. Ecdysone was implicated in this suppression by a reduction in *Broad Complex* expression and reduced pupation rates in r *Hb-ugt-1*-injected larvae. In addition to the finding that *H. bacteriophora* excreted-secreted products contain glycosyltransferase activity, these results demonstrate that *Hb-ugt-1* is an immunosuppressive factor and that its activity likely involves the inactivation of ecdysone.

Baculoviruses that infect insects, such as *Autographa californica*, have been shown to express a UDP-glycosyltransferase (UGT) that acts as a virulence factor through interference with ecdysone signaling^1^. The baculovirus UGT is believed to have been acquired from *Lepidopteran* spp. through horizontal gene transfer^2^, which implies that it should be ideally suited for activity in an insect host. During an infection, the *A. californica* UGT conjugates UDP-glucose present in the host with the ecdysteroid hormone 20-hydroxyecdysone (20E), effectively eliminating the molecule’s ability to activate its receptor^3^. Because 20E is the primary molting signal in insects, infected larvae will not molt, but instead continue to feed and accumulate mass that will be converted to higher viral output^4–6^. The set of effects controlled by 20E is broader than molting alone, however. Ecdysone has also been shown to regulate the immune response in *Drosophila melanogaster*^7^. In S2 cell cultures, 20E is known to enable Imd signaling and sensitize cells to the Toll pathway ligand Spätzle, in both cases promoting antimicrobial peptide (AMP) expression^8,9^.

Here, we examined a putative ecdysteroid glycosyltransferase Hba_07292 (*Hb-ugt-1*), from *Heterorhabditis bacteriophora*, that shares amino acid sequence similarity to baculovirus and insect UGTs. Further, we hypothesize that this glycosyltransferase functions in a similar manner to viral UGT virulence factors. A nematode UGT is unlikely to have been acquired by horizontal gene transfer, but nematodes do share an ecdysozoan lineage with insects, and therefore likely regulate molting in a similar way^10^. Interestingly, evidence exists that 20E might even be the signaling molecule responsible for regulating molting in a number of helminths. In the filarial parasite *Brugia malayi*, 20E elicits a transcriptional response featuring genes related to embryogenesis, and a homolog of the insect ecdysone receptor has been identified in its genome^11^. The genome of the nematode *Pristionchus pacificus* also contains a putative ecdysone receptor homolog, and another has been postulated in *H. bacteriophora*, though this has yet to be confirmed^12^. If this is indeed the case, *H. bacteriophora* would require a negative regulatory element that is active against 20E to moderate its own development. Alternatively, this molecule could function as a virulence factor similar to that of baculoviruses if it were secreted and diminished host 20E signaling. It is currently not possible to indicate whether *Hb-ugt-1* participates in development, but the known relationships between nematodes and 20E does allow for a plausible explanation of why the *H. bacteriophora* genome would contain a UGT active against 20E.

To further examine whether *Hb-ugt-1* functions as a virulence factor, we performed a number of assays related to its expression and in vivo activity in the model host *D. melanogaster*. In agreement with the RNA sequencing assay that initially identified this glycosyltransferase^13^, *Hb-ugt-1* was upregulated by *H. bacteriophora* infective juveniles (IJs) in response to hemolymph from multiple insect species, including *D. melanogaster*, though interestingly not in response to the nematode’s symbiotic bacterium *Photorhabdus luminescens*. A recombinant version of *Hb-ugt-1* (r*Hb-ugt-1*) suppressed the upregulation of AMP gene expression following microinjection, as do total excreted-secreted (ES) products of *H. bacteriophora*^14^. ES products also contained in vitro glycosyltransferase activity. Expression of the transcription factor *Broad Complex* and molting ability were subsequently characterized following r*Hb-ugt-1* injection to determine whether ecdysone signaling might play a role in the observed AMP suppression. Both were significantly diminished, suggesting a decreased amount of active insect 20E after exposure to r*Hb-ugt-1*. Generally, this collection of evidence is consistent with a role for *Hb-ugt-1* as an ecdysone-inactivating virulence factor.

## Results

### *Hb-ugt-1* is upregulated in response to host factors

**Figure 1:**
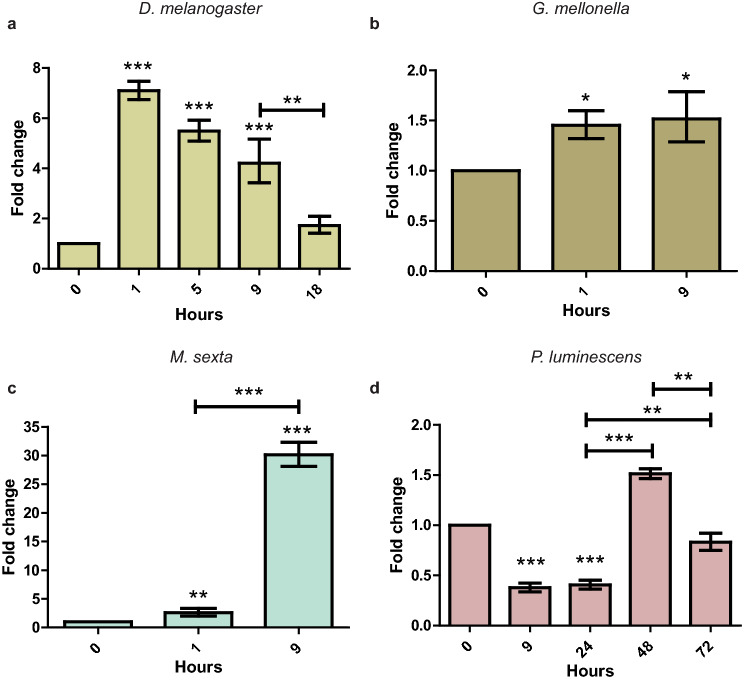
*Hb-ugt-1* is upregulated in response to host-associated factors, but not development-inducing symbiotic bacteria. The expression of *Hb-ugt-1* in response to assorted host factors and *Photorhabdus luminescens* bacteria was assessed by qPCR at the indicated time points. Approximately 1,000 axenic infective juveniles (IJs) were exposed to 25% *Drosophila melanogaster* line Oregon-R (**A**) or *Manduca sexta* (**C**) hemolymph plasma while 5,000 nematodes were injected directly into the hemocoel of live *Galleria mellonella* larvae (**B**). One thousand IJs were plated on a lawn of *P. luminescens* as a virulence-neutral control** (D**). Average fold change drawn from three independent trials and calculated according to the 2^−ΔΔCT^ method is shown, where error bars represent standard error (*p < 0.05, **p < 0.01, ***p < 0.001). Graphs were generated using GraphPad Prism version 5.03 (www.graphpad.com).

Originally, *Hb-ugt-1* was identified as part of the transcriptomic response to *Manduca sexta* hemolymph following a 9-h exposure^15^. To further explore the transcriptional response and examine expression in response to a variety of insect factors or cues, we measured *Hb-ugt-1* transcript levels in response to 25% hemolymph plasma from *D. melanogaster* and *M. sexta*, as well as an in vivo exposure to *Galleria mellonella* through injection of infective juveniles (IJs) directly into the hemocoel. Each of these treatments resulted in the upregulation of *Hb-ugt-1* between 1 and 9 h (Fig. 1A–C), indicating that the transcript is broadly upregulated in response to insect factors. Additionally, to distinguish between a role for the glycosyltransferase in infection rather than development, IJs were exposed to a lawn of *P. luminescens*. This treatment sustains the growth and development of *H. bacteriophora* but should not induce the expression of virulence factors if these are responsive strictly to insect host signals. Interestingly, *P. luminescens* induced a downregulation of *Hb-ugt-1* through 24 h, after which expression returned to a level comparable to 0-h expression (Fig. 1D). It should be noted as well that while expression of *Hb-ugt-1* is significantly higher at 48 h as compared to 24 h, the 48 h expression level is not statistically discernable from 0-h expression. Together, these results indicate that *Hb-ugt-1* is upregulated upon exposure to an insect host, but is unlikely to play a role in recovery of the nematode IJ from its diapause state.

### *Hb-ugt-1* contains a conserved ecdysteroid glycosyltransferase domain

**Figure 2:**
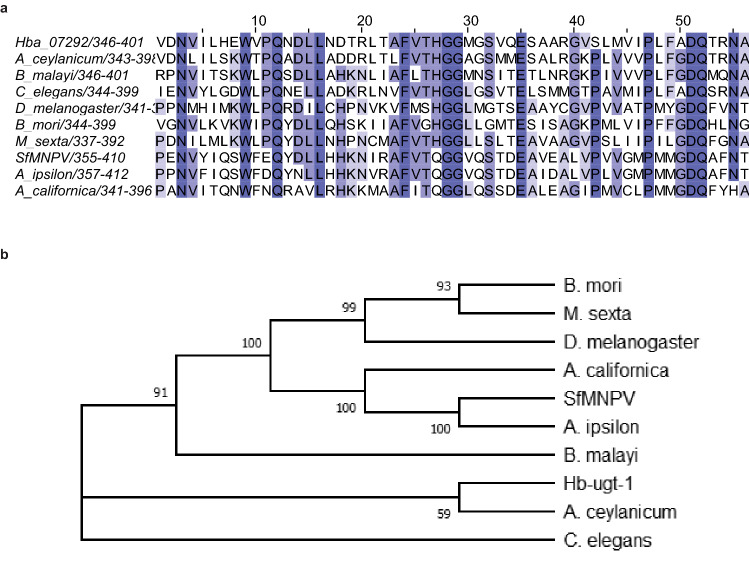
*Hb-ugt-1* is a putative UDP glycosyltransferase (UGT). (**A**) The amino acid sequence for *Hb-ugt-1* displays sequence similarity to ecdysone glycosyltransferases. A region containing the domain associated with sugar binding is shown, where color intensity represents percent identity. The full alignment was produced with sequences from three insects; *Drosophila melanogaster* (NP_001246082.2)*, Bombyx mori* (NP_001243972.1)*,* and *Manduca sexta* (XP_030029659.1), three nematodes; *Ancylostoma ceylanicum* (EYC08379.1)*, Brugia malayi* (XP_001894161.1), and *Caenorhabditis elegans* (NP_500913.1), and three insect-infective viruses; *Spodoptera frugiperda* multiple nucleopolyhedrovirus (AAP79109.1), *Agrotis ipsiolon* nucleopolyhedrovirus (YP_002268062.1), and *Autographa californica* nucelopolyhedrovirus (NP_054044.1). (**B**) A maximum-likelihood phylogenetic tree was generated for the aligned sequences with MEGA-X software. The consensus tree shown was developed from 200 bootstrap replicates and numbers next to branches indicate the percentage of replicates in which the associated taxa clustered together. The alignment image was produced with Jalview version 2.11.1.0 (www.jalview.org) and the image for the phylogenetic tree was exported from MEGA-X Version 10.1.5 (megasoftware.net).

Using BlastP search, we identified *Hb-ugt-1* as a putative glycosyltransferase containing an ecdysteroid glycosyltransferase domain (Interpro domain: IPR016224). An alignment of *Hb-ugt-1* with several related glycosyltransferases indicates a substantial degree of similarity between the proteins, especially with reference to the signature sequence associated with the binding of the nucleotide sugar (Fig. 2A). The maximum likelihood phylogenetic tree (Fig. 2B) for these sequences produces an intuitive clustering of insects, nematodes, and viruses. This is likely due to stretches of low-similarity in the full-length alignment (Supplementary Fig. S1) that are nonetheless more similar for species within the same phylum. Because of the marked conservation across relevant groups of organisms and the known function of the included viral UGTs, this was considered a sufficient justification for investigating whether *Hb-ugt-1* might function similarly.

### Activated *H. bacteriophora* excreted-secreted (ES) products facilitate glycosylation in the presence of 20-hydroxyecdysone and a UDP-glucose donor molecule

**Figure 3:**
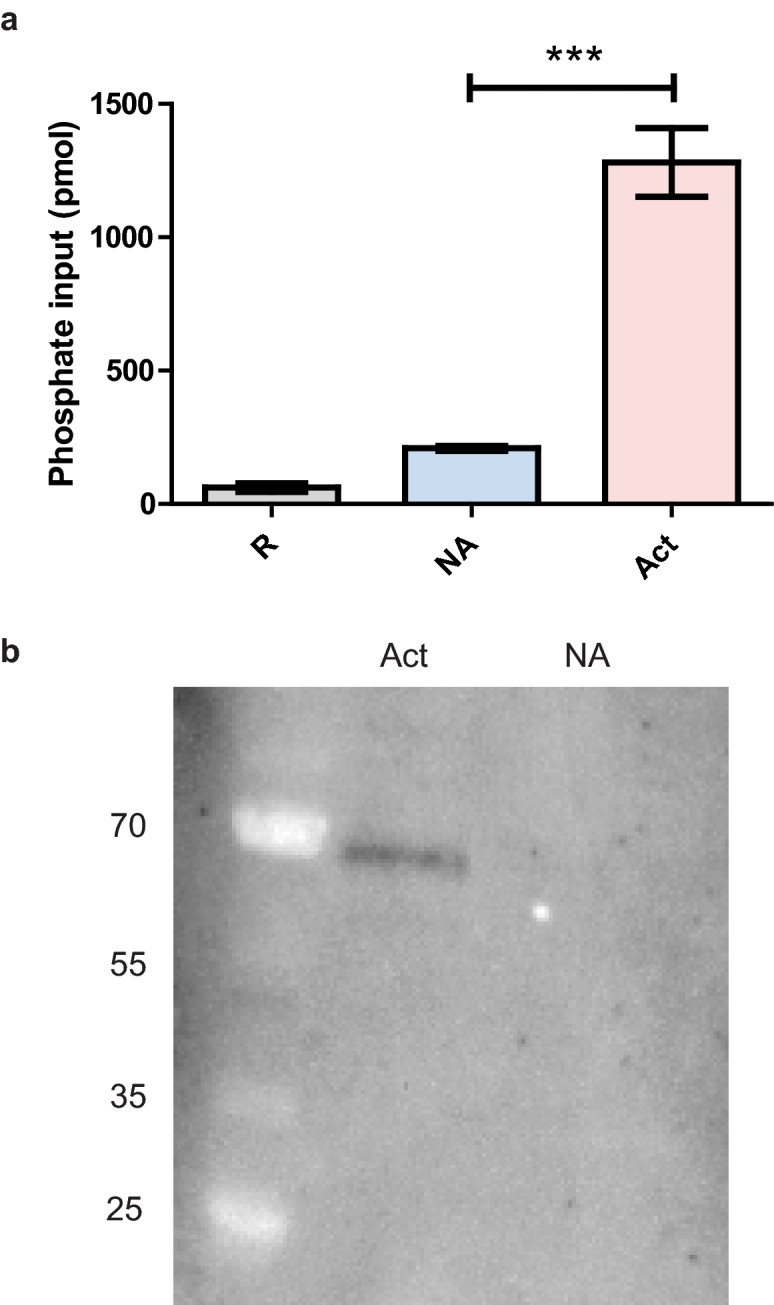
*Heterorhabditis bacteriophora* activated ES products glycosylate 20-hydroxyecdysone and contain a GTDC1 domain-containing protein. (**A**) Activated ES products (Act) were assayed for glycosyltransferase activity against Ringer’s Buffer (R) and non-activated ES product (NA) controls using a colorimetric assay. UDP-glucose and 20-hydroxyecdysone were used as the donor sugar and sugar acceptor, respectively. All values were normalized to a negative control containing only substrate and the manufacturer-provided reaction buffer. The experiment was performed in triplicate and results are presented as phosphate input liberated from the UDP molecule (***p < 0.001). (**B**) ES products were separated by SDS-PAGE and labeled with anti-GTDC1 antibody to identify glycosyltransferases secreted by the nematode. The size in kilodaltons (kDa) of ladder markers is indicated on the left side of the image. The phosphate output graph was produced with GraphPad Prism version 5.03 (www.graphpad.com).

The presence of a secretion signal at the N-terminus of *Hb-ugt-1*, as determined by SignalIP-5.0, suggests that the molecule may be secreted. Therefore, ES products of hemolymph-activated IJs were assessed for glycosyltransferase activity using a commercially available assay that detects phosphate liberated from the UDP donor molecule (UDP-glucose) following transfer of the UPD sugar to an acceptor molecule, which was 20E in this case. Glycosyltransferase activity was significantly higher (*p* < 0.001) in activated products as compared to both non-activated products and a Ringer’s buffer control (Fig. 3A). As a negative control, no signal was detected from activated products in the absence of 20E substrate. To further confirm the presence of a glycosyltransferase, the activated products, which contain a variety of proteins^14^, were assayed by western blot with a polyclonal antibody that recognizes human GTDC1 (Fig. 3B). A single band of approximately 60–70-kDa was detected in these activated products, consistent with the approximately 60-kDa molecular weight calculated from the *Hb-ugt-1* predicted protein sequence (Fig. 3B). Additionally, the GTDC1 antibody was assessed for its ability to bind r*Hb-ugt-1*, and this western blot was found to be positive (Supplementary Fig. S2). These results indicate that *H. bacteriophora* secretes a glycosyltransferase when exposed to an insect host, and that the activity is likely the result of a single protein, but more work is required to confirm that this protein is *Hb-ugt-1*.

### Recombinant *Hb-ugt-1* suppresses the upregulation of antimicrobial peptide (AMP) genes

**Figure 4:**
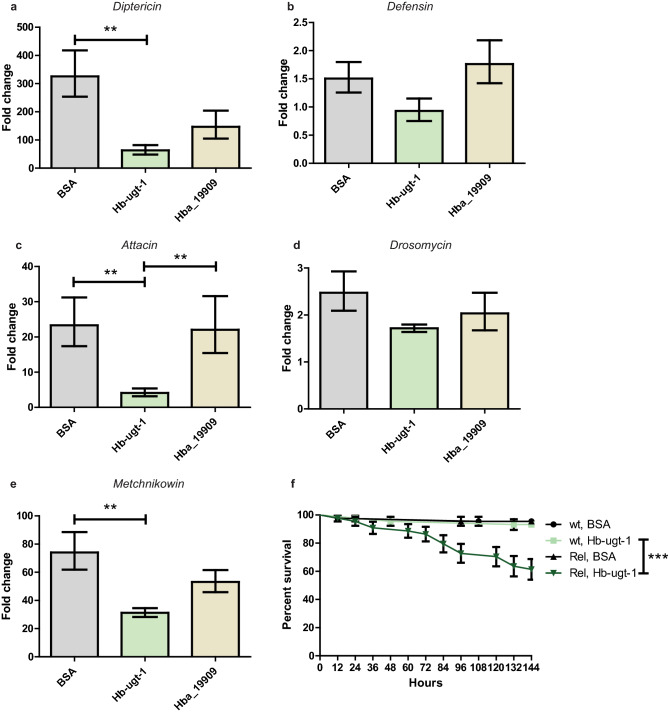
Recombinant *Hb-ugt-1* suppresses a subset of antimicrobial peptide genes in *Drosophila melanogaster*. (**A–E**) Adult flies of the Oregon-R line of *D. melanogaster* were injected with approximately 7 ng of recombinant protein or BSA prior to homogenization and RNA extraction at 6-h post injection. Expression for the indicated antimicrobial peptide genes was normalized to *rp49* and fold change was calculated relative to the 0-h time point. Average expression for three trials is shown where each trial consisted of two replicates with five flies each, three males and two females. Error bars indicate standard error and significance was assessed using a one-way ANOVA (**p < 0.01). (**F**) Alternatively, survival for injected *Rel*^*E20*^ (Rel) flies was assessed every 12 h and compared to that of the wild type (wt) *w*^*1118*^ line that serves as their genetic background. Curves consist of average values for three trials at two replicates of 10 flies each per trial. Bars represent standard error and significance was assessed with a Mantel–Cox test (***p < 0.001). All graphs were generated with GraphPad Prism version 5.03 (www.graphpad.com).

Recombinant *Hb-ugt-1* produced via an Sf9 expression system was used to examine immune-related effects in *D. melanogaster*. Adult flies injected with approximately 7 ng of r*Hb-ugt-1* or BSA in PBS were collected at 6-h post-injection to determine the expression of AMP genes representing both the Toll and Imd pathways by qPCR analysis. The *H. bacteriophora* putative i-type lysozyme rHba_19909, which was expressed and purified according to the same protocol as *Hb-ugt-1*, and BSA were injected separately as controls. The upregulation of *Diptericin, Attacin,* and *Metchnikowin* were all significantly lower following an injection of r*Hb-ugt-1* as compared to BSA, whereas injection of rHba_19909 had no effect (Fig. 4A,C,E). Although Hba_19909 does appear to have a mild inhibitory effect on the upregulation of *Diptericin* as compared to BSA, this difference was not statistically significant (*p* = 0.08).

To further examine the physiological significance of the r*Hb-ugt-1*-reduced AMP gene expression, r*Hb-ugt-1* was injected into *D. melanogaster Relish* mutants, which are incapable of mounting an Imd-based response due to a lack of the terminal transcription factor in the Imd pathway^15^. While these mutants were capable of surviving a BSA injection at a rate comparable to wild type, an injection of r*Hb-ugt-1* resulted in significantly lower survival over a six-day period (Fig. 4F). Conversely, wild type flies injected with r*Hb-ugt-1* survived at a similar rate as buffer-injected controls. The suppressed AMP upregulation demonstrates that r*Hb-ugt-1* has immunosuppressive effects, and the mortality seen in the recombinant-injected mutants indicates that the degree of this immunosuppression is consequential to the survival of the insect.

### *Hb-ugt-1* suppresses the expression of broad-complex, an ecdysone-responsive transcription factor

**Figure 5:**
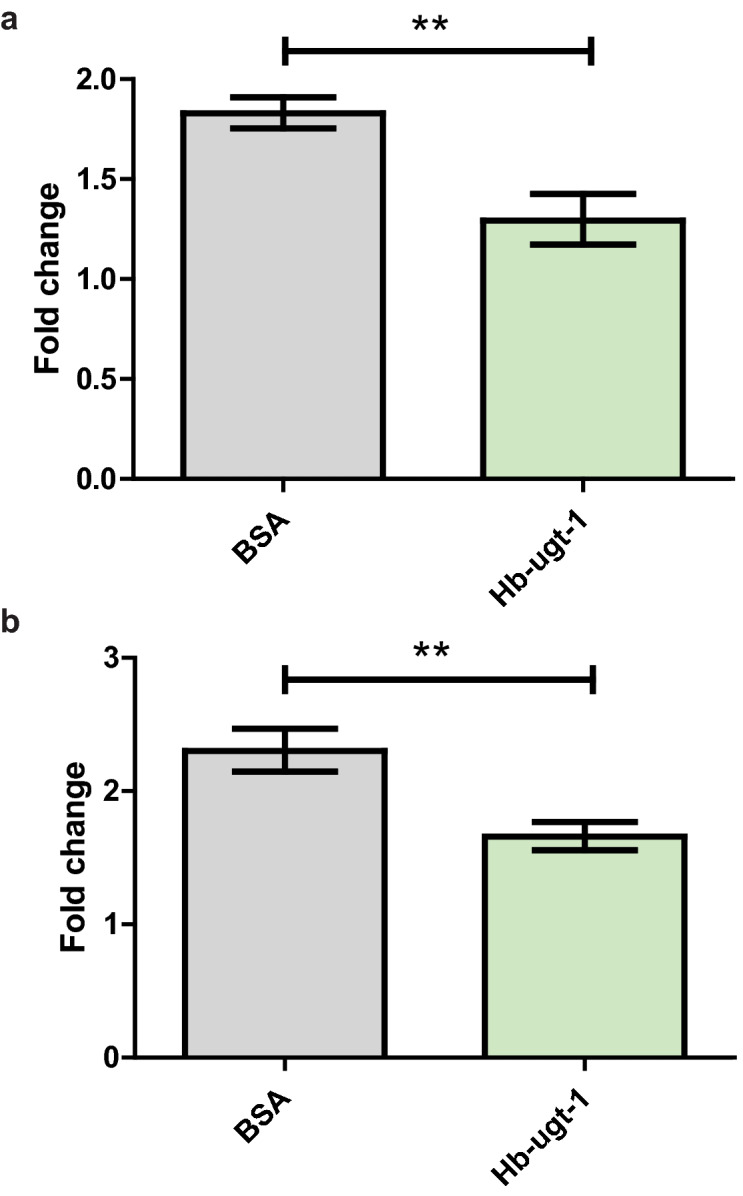
*Hb-ugt-1* recombinant protein suppresses Broad-Complex expression in *Drosophila melanogaster* adults and larvae. (**A**) Adult *D. melanogaster* Oregon-R were injected with approximately 7 ng of recombinant *Hb-ugt-1* or BSA and assessed for *Broad-Complex* expression normalized to *rp49* at a 6-h post injection. (**B**) Third instar larvae of the same line were co-injected with approximately 5 ng of recombinant protein and 1.5 ng of 20-hydroxyecdysone prior to collection at 0.5-h for RNA extraction and qPCR. Fold change relative to the 0-h time point is shown for three independent trials consisting of 10 larvae total across two replicates. Error bars indicate standard error and significance was assessed using a student’s t-test (**p < 0.01). Graphs were exported from GraphPad Prism version 5.03 (www.graphpad.com).

The binding of 20E to its receptor is known to induce the expression of several transcription factors, including *Broad-Complex* (*Br-C*), which subsequently upregulates components of the immune response, including the Peptidoglycan Recognition Protein LC (PGRP-LC) and some AMPs, even in the absence of Imd pathway activity^8^. To assess whether ecdysone signaling might be impaired in r*Hb-ugt-1*-injected *D. melanogaster*, larvae and adult flies were injected with 5 ng and 7 ng of r*Hb-ugt-1*, respectively. They were then processed for *Br-C* expression measurements via qPCR (Fig. 5). In both cases, *Br-C* expression was significantly reduced, at a 6-h time point in adult flies (Fig. 5A) and at a 30-min time point in larvae (Fig. 5B). Because of the closely associated transcriptional response of *Br-C* to 20E signaling, this suggests that 20E signaling is diminished in insects injected with the recombinant protein. This reduction may also be responsible for the decreased upregulation of AMPs seen in response to injection of r*Hb-ugt-1*, as disruption of *Br-C* alone has been found to be sufficient for a significant reduction in upregulation of AMPs, including *Diptericin*^8^.

### Pupation is delayed in larvae injected with r*Hb-ugt-1*

**Figure 6:**
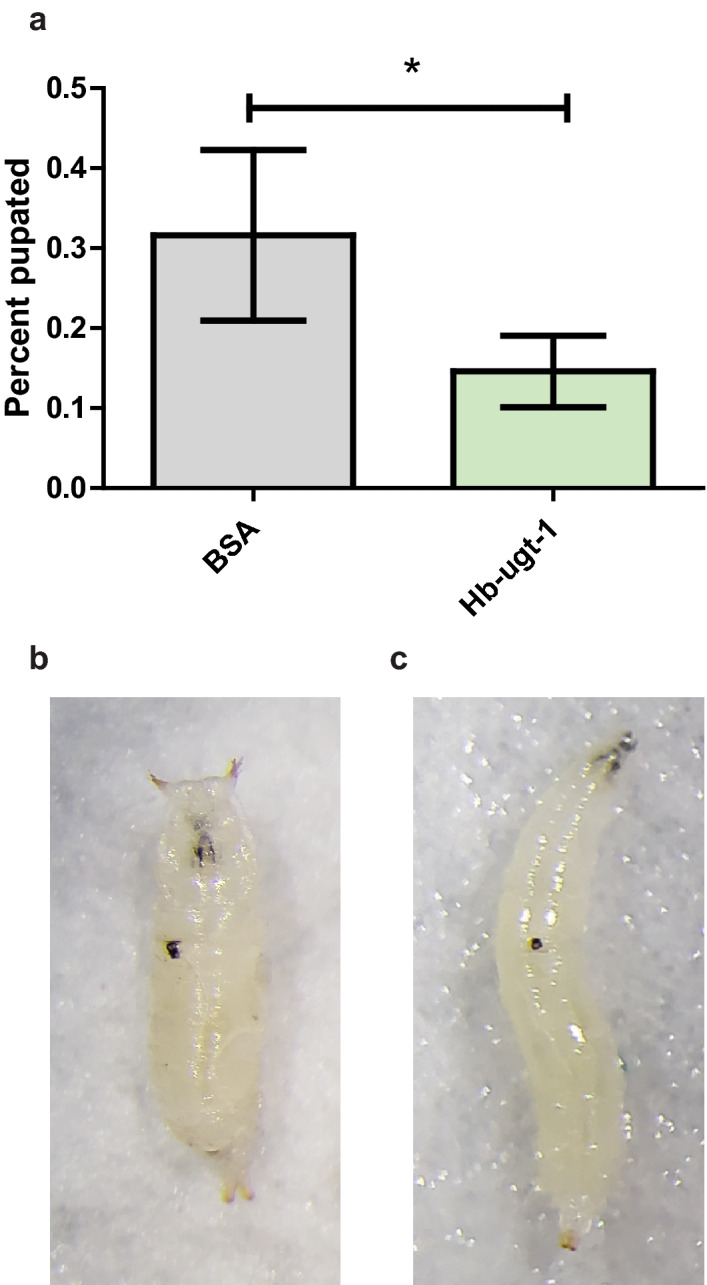
*Hb-ugt-1* reduces the pupation rate of third instar *Drosophila melanogaster* larvae. (**A**) Third instar larvae of Oregon-R *D. melanogaster* injected with 5 ng of recombinant *Hb-ugt-1* or BSA were surveyed at 8-h post-injection for immobility and spiracle inversion, which were used to indicate a commitment to pupation. Significantly fewer larvae begin pupation when injected with r*Hb-ugt-1* as compared to BSA. (**B** and **C**) Representative images are shown for a pupated larva featuring the characteristic spiracle inversion and a larva that has not begun pupation, respectively. Melanization is present at the wounding site for both larvae. Bars indicate the average rate collected from five independent trials and a total of 73 and 77 larvae, respectively, for BSA and r*Hb-ugt-1* injections. Error bars represent standard error and significance was assigned by Chi-Square analysis (*p < 0.05). The graph was generated using GraphPad Prism version 5.03 (www.graphpad.com).

To support the observed effect on *Br-C* expression, we injected *D. melanogaster* third-instar larvae (Fig. 6C) with 5 ng of r*Hb-ugt-1* or BSA and measured the timing of their commitment to pupation, as this process is regulated positively by ecdysone signaling. Commitment to pupation, based on immobility and spiracle inversion (Fig. 6B), was found to be approximately 50% lower following injection of r*Hb-ugt-1* as compared to BSA (Fig. 6A) at 8-h post injection. The total rate of pupation at a 24-h time point and the rate of eclosion were unaffected (Supplementary Fig. S3). This effect on pupation further implicates ecdysone signaling as a target of *Hb-ugt-1*. The fact that pupation is delayed, but not disabled also agrees with the prediction that a transient injection of r*Hb-ugt-1* will temporarily decrease the concentration of active 20E signaling hormone.

## Discussion

Based on its protein sequence, the candidate virulence factor gene *Hb-ugt-1* was predicted to possess a conserved ecdysteroid glycosyltransferase domain. This by itself does not necessarily indicate that this glycosyltransferase functions as a virulence factor, as glycosyltransferases are involved in a wide variety of processes^16^. Still, targeting ecdysone signaling with a glycosyltransferase is apparently such an effective strategy for undermining a host that most baculoviruses maintain an ecdysteroid UGT in their genome^17^. With this in mind, the possibility of a similar role for *Hb-ugt-1* warrants further investigation.

Typically, pathogens upregulate the expression of their virulence factors in response to host entry or physiological cues. Notably, we found that *Hb-ugt-1* gene expression is rapidly increased and sustained in response to insect hemolymph, both in vitro and in vivo. The magnitude of this increase varied between treatments, but this is to be expected given that the degree of IJ activation in other entomopathogenic nematodes depends on a collection of factors, including host species^18,19^. Each treatment showed significant upregulation by one hour, which by itself indicates that *Hb-ugt-1* could be involved in either virulence or the growth and development of the nematode, although the latter is made less likely by the observed downregulation following exposure to *P. luminescens* bacteria. Interestingly, the expression level of *Hb-ugt-1* on *P. luminescens* returns to a level comparable to the 0-h time point at 48 h. This time point is developmentally relevant in that this is approximately when *H. bacteriophora* nematodes will transition from recovering juveniles to fourth stage pre-hermaphrodites^20^ and at which point they will cease development without their symbiotic bacteria^21^. This developmental stage is also the point at which ecdysteroids reach their highest concentration in *Haemonchus contortus*^22^. An important avenue of future research would be to examine a potential role for UGTs in *H. bacteriophora* development and uncover the modes of regulation that make this possible.

Another important characteristic of a virulence factor is that it is secreted to the exterior of the nematode so that it can interact with the host environment, as documented in other species^23^. To this end, we tested *H. bacteriophora* ES products for glycosyltransferase activity using UDP-glucose as a sugar donor and 20E as the acceptor molecule, as these are the prevalent substrates for glycosyltransferases of invertebrates and plants^24,25^. Glycosyltransferase activity was observed only from the ES products of hemolymph-activated IJs, indicating that *H. bacteriophora* does produce and externally secrete a glycosyltransferase in response to host exposure. It should be noted, however, that while this assay does demonstrate glycosyltransferase activity, additional work is required to confirm 20E as the substrate due to the fact that glycosyltransferases can act on other proteins and lipids present in the ES products^26^. The presence of glycosyltransferase activity was further supported by the identification of a GTDC1-labeled protein in the activated ES products of *H. bacteriophora*. While this protein is larger than the predicted size, it is within *Hb-ugt-1* a range that could be attributed to post-translational modification. Other helminths also produce multiple isoforms of UDP-glycosyltransferases^27^, which may be the case with *H. bacteriophora* and could alternatively explain the size discrepancy.

To test the immunosuppressive capacity of the recombinant protein, we examined the induction of AMP encoding genes following injection of r*Hb-ugt-1*, where the tissue damage caused by injection alone is known to activate both the humoral and cellular immune responses^28,29^. The AMP genes *Diptericin*, *Attacin*, and *Metchnikowin* were upregulated to a significantly lower degree following r*Hb-ugt-1* injection as compared to BSA injections. *Drosomycin* and *Defensin* (Fig. 4B and D) were induced at comparable levels for each treatment, but this may be at least partially due to a lower baseline responsiveness to injection injury. In the case of *Defensin*, this AMP would also not be expected to have an impact on the course of an infection as neither *Steinernema* nor *Heterorhabditis* nematodes induce *Defensin* expression in *D. melanogaster* larvae^30,31^. With regard to toxicity, r*Hb-ugt-1* was not found to induce mortality in wild type flies, but the lower survival rate in immunocompromised flies injected with recombinant protein does indicate that the immune or otherwise physiological effects are relevant to the survival of the fly. An exact cause of mortality in these flies unfortunately cannot be determined from these data alone, but this question represents an important point to resolve in future work.

Two separate ecdysone-responsive factors were tested to determine if the immunosuppressive effect of r*Hb-ugt-1* was related to ecdysone signaling. Expression of the transcription factor *Br-C* was selected due to its documented role as a link between ecdysone signaling and innate immunity^32–34^, and concordantly its expression was reduced in both *D. melanogaster* adults and larvae following injection of r*Hb-ugt-1*. Furthermore, a delay in the onset of pupation was observed in third-instar larvae injected with the recombinant, similarly indicating diminished ecdysone signaling, as 20E is the primary signal for this process^35^. This indicates that the *H. bacteriophora* glycosyltransferase may be acting in a manner similar to the baculovirus UGTs, namely that its observed effects appear to stem from a capacity to deactivate the 20E signaling hormone.

Additional work must be done to fully elucidate the role of *Hb-ugt-1* as a virulence factor with activity against 20E, but the findings presented here offer strong foundational support for this notion. Future work could expand on the information presented here by further clarifying the role of *Hb-ugt-1* as it pertains to development within the nematode as well as virulence, and also the collection of immune impacts *Hb-ugt-1* might have, given that ecdysone is involved in a variety of immune mechanisms, including the nodulation response^36,37^. The in vitro reaction properties of the recombinant enzyme may also be examined to investigate how this protein participates in the dynamics of a natural infection. These questions are of great interest for the description of host-parasite interactions, but this knowledge could have broader impacts as well. Information about individual virulence factors can be used to develop stronger parasites for the biocontrol of insect pests, which is a widespread application of *Heterorhabditis* species^38^, and to combat nematode virulence strategies, as steroid hormones likewise play a role in vertebrate immunity^39^, and may be targeted by glycosyltransferases of vertebrate-infective nematode parasites.

## Methods

### Infective juvenile activation for *Hb-ugt-1* expression analysis

For in vitro activation experiments in insect hemolymph, 20 third-instar *D. melanogaster* larvae were collected, rinsed with water, and pinched with forceps near the mouth hooks to create a small incision. Before releasing the forceps, larvae were submerged in a 5 μl aliquot of a 2.5 μg/ml solution of phenylthiourea (PTU) in PBS, contained in a 0.5 ml centrifuge tube nested in a 1.5 ml centrifuge tube. Critically, the 0.5 ml tube featured an incision at the bottom of the tube allowing for passage of fluid, but not larvae from the 0.5 ml tube to the 1.5 ml tube. Hemolymph was collected from the larva by centrifugation at 4 °C for 30 s at 17,900 × *g*. The resulting approximately 10 μl of extracted hemolymph was diluted two-fold with PTU-PBS, filtered with a 10 μm polyethylene filter, and kept on ice to prevent melanization. This process was repeated three times to produce a sufficient volume of hemolymph for each activation trial. To collect *Manduca sexta* hemolymph, the posterior horn of a fifth instar larvae was surface sterilized with 70% ethanol and cut to release the hemolymph. The extracted hemolymph was diluted 1:4 in ice-cold Ringer’s buffer (100 mM NaCl, 1.8 mM KCl, 2 mM CaCl_2_, 1 mM MgCl_2_, and 5 mM HEPES, pH 6.9) supplemented with PTU to a final concentration of 0.33 mM. Before use, *M. sexta* hemolymph was centrifuged for 5 min at 4,000 × *g* and filtered with a 0.45 μm syringe filter. Approximately 1,000 *H. bacteriophora* IJs were transferred to a 1.5 ml centrifuge tube and washed twice with water by centrifugation for 30 s. After the second wash, the supernatant was discarded and a 40 μl aliquot of the extracted hemolymph solution was pipetted into the pellet of IJs and mixed before incubating at 28 °C for the specified time points. Axenic IJs for these experiments were generated as previously described^14^, where IJs were propagated in fifth or sixth instar *G. mellonella* larvae that had been infected with the RET16 derivative of *P. temperata* NC1.

In vivo activation of IJs was carried out by the injection of IJs directly into *G. mellonella* larvae. The injected aliquot of IJs was prepared by pelleting 5,000 nematodes as in the in vitro experiments, though with the addition of a 5-min axenization step using a 3% hypochlorite solution. These surface-sterilized IJs were washed three times with PBS and concentrated into a 100 μl volume of PBS. Nematodes were injected into *Galleria* larvae using an 18.5G needle and 1 ml syringe after the larvae had been surface-sterilized with 70% ethanol and anesthetized on ice for 15 min. The injected larvae were returned to the ice for a period of five minutes before being transferred onto slightly moist filter paper in a petri dish. After the allotted incubation time, the injected *Galleria* were dissected in PBS and an average of approximately 2,000 IJs were collected from the solution by pipette.

Gene expression changes in response to *P. luminescens* exposure were measured by directly exposing *H. bacteriophora* IJs to a bacterial lawn. *Photorhabdus luminescens* subspecies *laumondii*, strain TT01, was grown overnight in LB at 28 °C with shaking at 220 rpm. A 50 μl aliquot of overnight culture was spread in the center of LB agar plates, and the plates were incubated for 48 h at 28 °C. Approximately 2,000 washed *H. bacteriophora* IJs in a 100 μl aliquot of water were applied to the *P. luminescens* lawn and allowed to incubate at room temperature in the dark on a moist paper towel until the desired time point. In order to recover nematodes from the plate, 5 ml of M9 buffer were pipetted directly onto the plate and swirled gently before being collected and dispensed into a 15 ml Falcon tube. This process was performed three times in total for each plate to ensure optimal recovery.

### Sequence alignment and phylogenetics

The sequence alignment was produced with BioEdit software (version 7.2.5) following the ClustalW algorithm and organized graphically with JalView software (version 2.11.0). The gap opening and extension penalties for pairwise alignments were set to 1 and 0.1, respectively, while the corresponding penalties for the multiple alignment were 3 and 0.2. Minor manual adjustment was permitted where the alignment could be improved unambiguously. The phylogenetic tree was constructed using MEGA software (version 10.1.5) according to the Maximum Likelihood method with 200 bootstrap replicates.

### Glycosyltransferase activity in *H. bacteriophora* secreted products

Excretory-secretory products were collected from axenic *H. bacteriophora* IJs as described previously^14^. Briefly, 200,000 IJs were soaked in 25% *M. sexta* hemolymph for a period of 20 h, washed in Ringer’s buffer, and placed in fresh Ringer’s buffer for a 5-h ES product collection period. The resulting supernatants were filtered and concentrated such that supernatants from three preparations of 200,000 IJs each were pooled and concentrated to a final volume of 100 μl. This allowed standardization of ES products to equal numbers of IJs so that despite activated and non-activated products containing different concentrations of protein, they both contain 2,000 IJ equivalents/μl. Glycosyltransferase activity was assayed with a colorimetric assay specific for phosphate-coupled glycosyltransferase reactions (R&D Systems Glycosyltransferase Activity Kit EA001) according to the manufacturer’s instructions. The glycosyltransferase activity of 10,000 IJ equivalents (5 μl) was measured using 5 mM UDP-glucose (Abcam) and 5 mM 20-hydroxyecdysone (Sigma) in DMSO as the sugar donor and acceptor for the reaction, respectively. Reactions were incubated at 37 °C for 3 h, at which point the samples were diluted with 100 μl of deionized water in a 96-well flat bottom plate (Greiner) and 30 μl each of the provided Malachite Green reagents were added sequentially. Each sample was incubated for an additional 20 min at room temperature before absorbance was measured at 620 nm. Absorbance measurements collected from three independent experiments were analyzed for statistical significance by one-way ANOVA.

### Protein electrophoresis and western blotting

As described previously^14^, the concentration of protein in activated ES products was determined using a Pierce BCA Protein Assay Kit (Thermo Scientific). As the activated products were the only preparation to produce a readable signal from the BCA assay, a 10 μl aliquot corresponding to 150 ng of activated products and an equivalent 10 μl volume of non-activated products were reduced in 50 mM DTT and loaded into a Novex WedgeWell 4–20% Tris–Glycine Gel (Invitrogen). The composition of each well was adjusted to 26 μl of sample and water, 4 μl of DTT, and 10 μl of Laemmli buffer. Additionally, 5 μl of PageRuler Plus Prestained Protein Ladder (Thermo Scientific) were loaded separately for size estimation. To label glycosyltransferases in the ES products, the gel was transferred to nitrocellulose with a Trans-Blot Turbo Transfer System (Bio Rad) and blocked overnight at 4 °C with 5% powdered milk in Tris-NaCl-Tween (TNT, 25 mM Tris pH 7.4, 0.5 M NaCl, 0.1% Tween 20)^40^. The membrane was then incubated with a 1:2,000 dilution of GTDC1 rabbit polyclonal antibody (Proteintech) in 5% powdered milk for 2 h at room temperature. Secondary antibody labeling was achieved with a 1-h incubation with a 1:2,000 dilution of anti-rabbit IgG, HRP-linked antibody (Cell Signaling Technology) in 5% dry milk at room temperature. Signal for the HRP substrate was generated by exposure to Supersignal West Femto Maximum Sensitivity Substrate (Thermo Fisher) for 5 min.

### Recombinant expression of *H. bacteriophora* candidate genes

Two candidate virulence factor genes were expressed in Sf9 cells using a pMIB/V5-His A expression vector. Initial amplicons were generated using primers specific for *Hb-ugt-1* (F: 5′ CTTGGTACCTAAAATCCTAGTCTTTAGCC 3′; R: 5′ AGACTCGAGTTCGGATTTCATTTTTTTCTCCG 3′) and Hba_19909 (F: 5′ CTTGGTACCCAATTGCCTTCATTGTATTTG 3′; R: 5′ AGACTCGAGCGAACAGCCACAGCACTTTTTGA 3′), each modified to contain restriction sites for KpnI and XhoI at the 5′ and 3′ ends, respectively, of the amplicons. Following cloning of the amplicons, One Shot TOP10 chemically competent *E. coli* were transformed with these constructs and selected on LB plates containing 100 μg/mL ampicillin. The same primers were used to confirm successful transformations via colony-PCR prior to growth in liquid culture, plasmid isolation, and sequencing. Plasmids featuring correct sequences were transfected into Sf9 insect cells using Cellfectin II reagent (Invitrogen) and selectively allowed to colonize wells in a 6-well plate by propagating in Sf-900 II SFM (Thermo Fisher) containing 10 μg/ml blasticidin and 15 μg/ml gentamycin. Upon sufficient growth and differentiation from untransfected cells, cultures were suspended in 300 ml of liquid medium and allowed to grow at 27 °C with shaking at 130 rpm until they reached a concentration of 6 × 10^6 ^cells/ml. Culture supernatants were queried for the presence of recombinant protein by western blot, targeting the V5 epitope (Anti-V5-HRP antibody, Invitrogen), and if positive, the total culture supernatants were prepared for purification, first through concentration driven by 8 kDa polyethylene glycol flakes, and then dialysis in 150 mM sodium phosphate at 4 °C for approximately 16 to 20 h. The resulting solution was centrifuged to remove debris, diluted 1:3 in low-stringency wash buffer (50 mM NaH_2_PO_4_, 500 mM NaCl, 40 mM Imidazole, 0.5% Tween 20), and co-incubated with 1 ml Ni-NTA agarose (Qiagen) for 1 h rotating at 90 rpm at room temperature. The recombinant-bound matrices were loaded onto an affinity purification column and washed with 10 equivalents of low-stringency wash buffer. Following this washing step, elution proceeded with two individual washes of high-stringency buffer (50 mM NaH_2_PO_4_, 500 mM NaCl, 100 mM imidazole, 0.5% Tween 20) followed by 6 individual washes with elution buffer (50 mM NaH_2_PO_4_, 300 mM NaCl, and 250 mM imidazole). Each aliquot was assessed again by western blot and those fractions containing recombinant protein were further concentrated against polyethylene glycol and dialyzed overnight in PBS. Before storage at − 20 °C, protein concentration was determined with a Pierce BCA Protein Assay Kit (Thermo Fisher) and the solution containing the purified recombinant protein was supplemented with protease inhibitor cocktail (Sigma).

### Injection of flies and larvae for the assessment of gene expression, survival, and pupation rate

All injections were performed with a Drummond Nanoject III Programable Nanoliter Injector, where treatments were transmitted through an oil-filled pulled glass capillary that had been opened with forceps to a degree that would allow delivery of the treatments while causing minimal damage to the organism. Adult flies were injected between 7 and 10 days after eclosion, whereas larvae were selected once they had reached the wandering third instar phase. In both cases, *D. melanogaster* stocks were maintained on a yeast—supplemented cornmeal-soy-based diet (Meidi Laboratories) at 25 °C on a 12-h day-night cycle. Oregon-R flies were used for AMP and *Br-C* expression studies while survival rates were collected in reference to the *Rel*^*E20*^ line and its associated background, *w*^*1118*^. All injections were preceded by a period of anesthetization with carbon dioxide, though generally the technique was adapted to optimize the recovery of each life stage. Adults were injected intramesothoracially with an approximately 69 nl aliquot containing 7 ng of recombinant protein or BSA in PBS-protease inhibitor solution before placement in vials containing instant *Drosophila* medium (Carolina Biological) incubated under the same conditions. Survival was assessed once every 12 h, or flies were collected at the 6-h time point for gene expression analysis. Alternatively, Oregon-R larvae were anesthetized with carbon dioxide for 2 to 3 min before being placed on slightly moist filter paper and injected with 5 ng of each treatment. Injections were delivered at a shallow angle between segments in the dorsal side of the abdomen, such that the probability of damaging the imaginal discs or organs would be low. Injected larvae were placed on fresh filter paper moistened with Ringer’s buffer and incubated at 25 °C for a period of 8 h, at which point larvae were observed for characteristics indicative of pupation.

### Gene expression analysis

As previously described^14^, RNA was extracted from five flies or larvae, or the entirety of the activated nematodes. Isolation was achieved via homogenization in TRIzol reagent (Ambion, Life Technologies), where IJs were freeze-thawed at − 80 °C for four repetitions prior to pestle homogenization to enhance yield. Reverse transcription was performed on 1 μg of total RNA using a High-Capacity cDNA Reverse Transcription Kit (Applied Biosystems). Quantitative PCR (qPCR) reactions were conducted and monitored with a CFX96 Real-Time System, C1000 Thermal Cycler (Bio-Rad) with the following cycling conditions: 95 °C for 2 min, 40 repetitions of 95 °C for 15 s followed by 61 °C for 30 s, and then one round of 95 °C for 15 s, 65 °C for 5 s, and finally 95 °C for 5 s. Each reaction well contained 10 μl GreenLink No-ROX qPCR Mix (BioLink), 40 ng of cDNA template, forward and reverse primers at a final concentration of 200 nM and ultrapure water to 20 μl total. Primers were as follows: *Diptericin* (F: 5′ GCTGCGCAATCGCTTCTACT 3′; R: 5′ TGGTGGAGTTGGGCTTCATG 3′), *Defensin* (F: 5′ CGCATAGAAGCGAGCCACATG 3′; R: 5′ GCAGTAGCCGCCTTTGAACC 3′), *Drosomycin* (F: 5′ GACTTGTTCGCCCTCTTCG 3′; R: 5′ CTTGCACACACGACGACAG 3′), *Metchnikowin* (F: 5′ TCTTGGAGCGATTTTTCTGG 3′; R: 5′ AATAAATTGGACCCGGTCTTG 3′), *Attacin* (F: 5′ CAATGGCAGACACAATCTGG 3′; R: 5′ ATTCCTGGGAAGTTGCTGTG 3′) *Hb-ugt-1* (F: 5′ TTCTTAACGACACGCGACTG 3′; R: 5′ CTCGTGCTGCAGATTCTTGA 3′), *Broad-Complex* (F: 5′ GAGCACACCCTGCAAACAC 3′; R: 5′ GCTGCGTGAGTCCAGAGAC 3′), *rp49* (F: 5′ GATGACCATCCGCCCAGCA 3′; R: 5′ CGGACCGACAGCTGCTTGGC 3′), and *rpl32* (F: 5′ ATCGGATAGATACCACCGCC 3′; R: 5′ TTGTGGGCATAGCACG 3′).

Statistical analysis for gene expression was performed on values derived from qPCR measurements processed according to the 2^−ΔΔC^_T_ method^41,42^ with all values being normalized to *rp49* in the case of flies and *rpl32* for *H. bacteriophora*. The expression of *Hb-ugt-1* in *H. bacteriophora* was assessed using a one-way ANOVA comparing dCt values gathered from three independent experiments. A Bonferroni multiple comparisons test was used to assign significance to comparisons between specific treatments. For all other gene expression assays, ddCt values were collected from three independent trials at two replicates per trial. Antimicrobial peptide gene expression was assessed with a one-way ANOVA as above while *Broad-Complex* expression was analyzed with a student’s t-test.

## Supplementary information

Supplementary information.
