# Supplementary material for: A putative UDP-glycosyltransferase from Heterorhabditis bacteriophora suppresses antimicrobial peptide gene expression and factors related to ecdysone signaling
Source: Sci Rep. 2020 Jul 23;10:12312. doi: 10.1038/s41598-020-69306-2 (PMC7378173; doi:10.1038/s41598-020-69306-2)

A putative UDP-glycosyltransferase from *Heterorhabditis bacteriophora* suppresses antimicrobial peptide gene expression and factors related to ecdysone signaling

Eric Kenney, Amulya Yaparla, John M. Hawdon, Damien M. O' Halloran, Leon Grayfer, Ioannis Eleftherianos

Supplementary Information

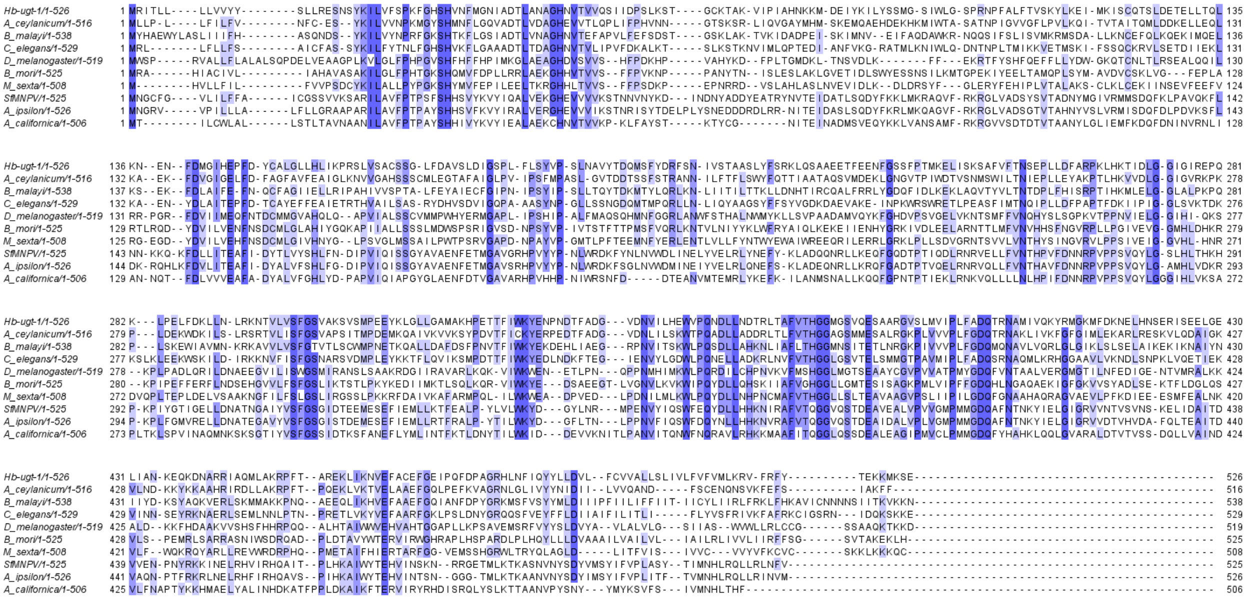

Figure S1. *Hb-ugt-1* shows sequence similarity to other ecdysone glycosyltransferases. The full-length alignment associated with the excerpt in figure 2 is shown. Color intensity represents percent identity.

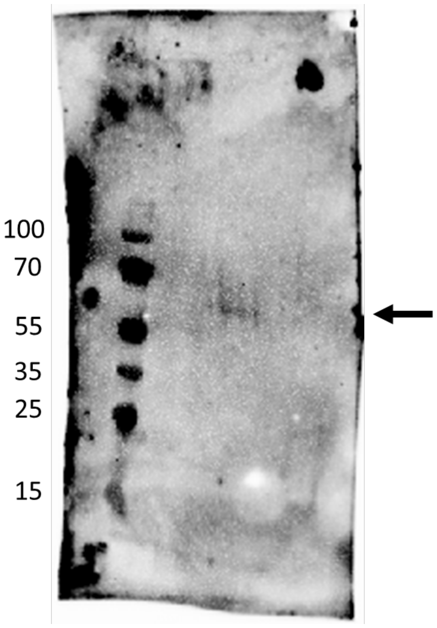

Figure S2. The GTDC1 antibody labels r*Hb-ugt-1*. An aliquot of approximately 1.8 µg of the recombinant protein was separated on an SDS-PAGE gel and subsequently labeled with anti-GTDC1 antibody via western blot. The arrow indicates the migration distance for the recombinant protein, which correlates to its predicted size of 59.5-kDa. Ladder sizes are indicated in kDa on the left side of the image.

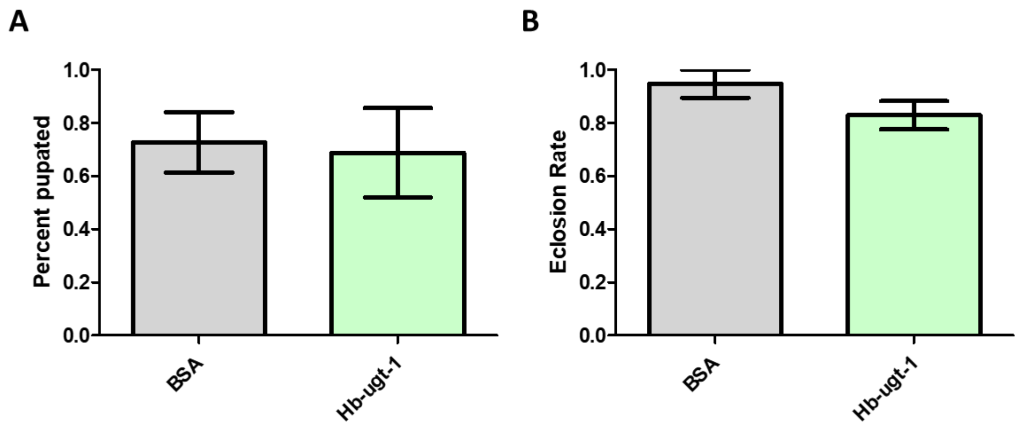

Figure S3. Injection with recombinant *Hb-ugt-1* does not affect total pupation or eclosion rate in *Drosophila melanogaster.* Third instar *Oregon-R D. melanogaster* larvae injected with 5 ng of r*Hb-ugt-1* or BSA were monitored for pupation at 24 hours and then daily for eclosion. No significant differences were observed between the two treatments as assessed by Chi-Square analysis.

Figure 3B (uncropped western blot)

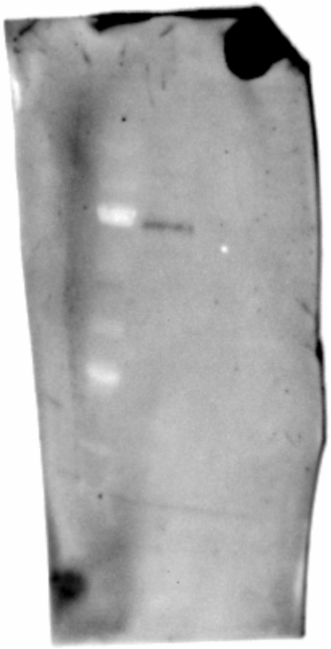

Supplement: Supplementary file 1 — Supplementary information. [file 41598_2020_69306_MOESM1_ESM.pdf]
